# Aspochalasin H1: A New Cyclic Aspochalasin from Hawaiian Plant-Associated Endophytic Fungus *Aspergillus* sp. FT1307

**DOI:** 10.3390/molecules26144239

**Published:** 2021-07-12

**Authors:** Mallique Qader, KH Ahammad Uz Zaman, Zhenquan Hu, Cong Wang, Xiaohua Wu, Shugeng Cao

**Affiliations:** 1 Department of Pharmaceutical Sciences, Daniel K. Inouye College of Pharmacy, University of Hawai’i at Hilo, Hilo, HI 96720, USA; mqader@hawaii.edu (M.Q.); kzaman@hawaii.edu (K.A.U.Z.); congwang@hawaii.edu (C.W.); xiaohua3@hawaii.edu (X.W.); 2 Warshel Institute for Computational Biology, The Chinese University of Hong Kong, Shenzhen 518172, China; huzhenquan@cuhk.edu.cn; 3 School of Chemistry and Materials Science, University of Science and Technology of China, Hefei 230026, China; 4 Guangxi Key Laboratory of Chemistry and Chemical Engineering of Forest Products, School of Chemistry for Nationalities, Nanning 530006, China

**Keywords:** endophytic fungi, *Aspergillus*, aspochalasin, epoxide, antibacterial activity

## Abstract

*Aspergillus* is one of the most diverse genera, and it is chemically profound and known to produce many biologically active secondary metabolites. In the present study, a new aspochalasin H1 (**1**), together with nine known compounds (**2**–**10**), were isolated from a Hawaiian plant-associated endophytic fungus *Aspergillus* sp. FT1307. The structures were elucidated using nuclear magnetic resonance (NMR) (^1^H, ^1^H-^1^H COSY, HSQC, HMBC, ROESY and 1D NOE), high-resolution electrospray ionization mass spectroscopy (HRESIMS), and comparisons with the reported literature. The absolute configuration of the new compound was established by electronic circular dichroism (ECD) in combination with NMR calculations. The new compound contains an epoxide moiety and an adjacent *trans*-diol, which has not been reported before in the aspochalasin family. The antibacterial screening of the isolated compounds was carried out against pathogenic bacteria (*Staphylococcus aureus*, Methicillin-resistant *S. aureus* and *Bacillus subtilis*). The antiproliferative activity of compounds **1**–**10** was evaluated against human breast cancer cell lines (MCF-7 and T46D) and ovarian cancer cell lines (A2780).

## 1. Introduction

Searching for antimicrobial agents has become one of the major research fields in natural products chemistry [1,2] because the development of pathogens resistant to the clinically used antimicrobials has been increasing alarmingly day by day [3,4]. Therefore, the usage of initially discovered antibiotics and antifungal drugs in treating human diseases has been greatly reduced [5]. Hence, there is an urgent need for new antibiotics in order to tackle, in particular, antimicrobial resistance. The accidental discovery of first broad spectrum antibiotic penicillin from the fungus *Penicillium notatum* has marked the starting point of the antibiotic’s era [6]. The diversity and the distribution of the fungi in a diverse array of habitats categorized them as an understudied group of organisms [7]. Fungi are considered remarkable and valuable organisms as they have provided the world with medicinally important drugs such as antibiotics, anticancer agents, cholesterol lowering statins, immunomodulators, and agriculturally important herbicides and weedicides, and they are environmentally important as decomposers and for the distribution of nutrients [8,9,10,11].

Fungi harbored in untapped and unique environments produce new compounds with greater biological activity. Endophytic fungi that inhabit plant tissues are a widely studied group of organisms as they live inside the plant tissues without having any adverse effect on the plants [8,9,10,11]. Additionally, they are capable of biosynthesizing similar chemicals which are produced by the plant or new compounds that plants could not synthesize by themselves [12]. Therefore, endophytic fungi have recently become popularized in the field of drug discovery following the discovery of the million-dollar anticancer drug Taxol from the fungal endophyte *Taxomyces andreanae*, which was isolated from the inner bark of the Pacific yew, *Taxus brevifolia* [13].

*Aspergillus* is one of the dominant and most studied fungal genera in endophytes [14]. The World Data Centre for Microorganisms (WDCM) has reported around 380 species in the *Aspergillus* genus [14]. More than 350 new fungal metabolites have been discovered during 2015–2019 [14]. Therefore, these filamentous fungi are described as one of the richest sources of bioactive fungal secondary metabolites with a great chemical and biological diversity [14,15].

Since the diversity and abundance of fungi are excessive, their presence in different ecosystems makes them an ideal source of drug discovery [7]. Hawai’i has 10 out of 14 world climatic zones. These diverse climatic, ecological and geographical features have made Hawai’i a home for organisms with a vast diversity. Our studies over the past few years disclosed that fungi isolated from different Hawaiian sources such as soil, plants and the marine environment showed a great chemical and biological diversity [16,17,18,19,20,21,22,23,24].

**Figure 1:**
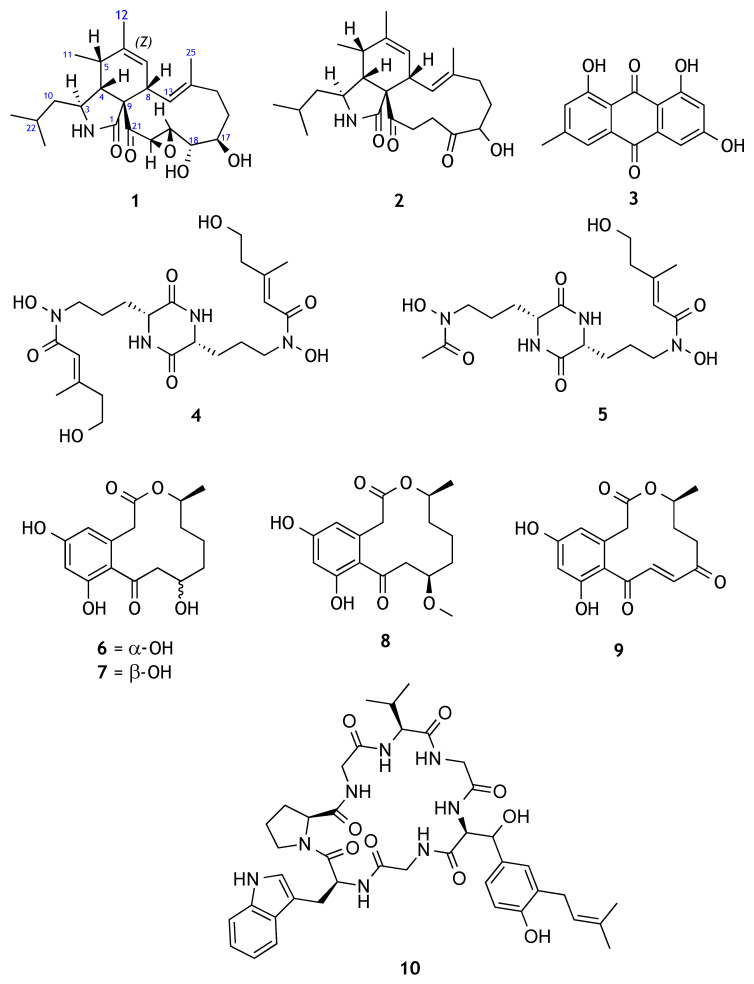
Structures of compounds **1**–**10**.

As part of our continuing search for new and biologically active compounds from Hawaiian fungi, six fungal endophytes were isolated from the leaves of *Heliotropium* sp. (family Boraginaceae) (http://theplantlist.org, accessed on 28 June 2021). The crude extracts obtained from a small-scale fermentation of these fungi were evaluated for their antibacterial activity, but only FT1307 showed inhibition against the methicillin-resistant *Staphylococcus aureus* (MRSA) and *Escherichia coli* (at 80 μg/mL). This observation led us to further study the fungal strain *Aspergillus* sp. FT1307 and to identify the antimicrobial compounds. A new cyclic aspochalasin (**1**) together with nine known fungal metabolites (**2**–**10**) (Figure 1) were isolated, and their structures were determined. Herein we report the large-scale fermentation, isolation and structure elucidation of the fungal metabolites and their antimicrobial activity against a panel of pathogenic microorganisms, together with their antiproliferative activity.

## 2. Results and Discussion

**Figure 2:**
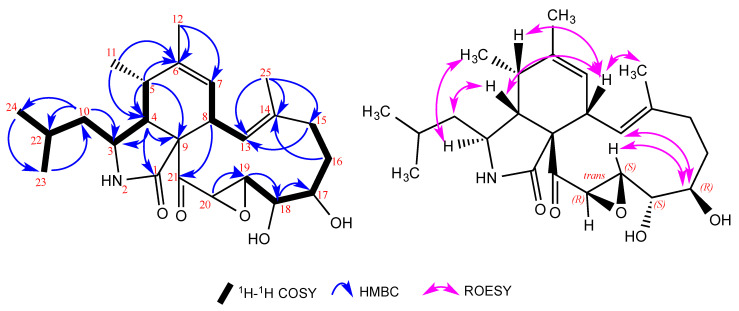
^1^H-^1^H COSY, HMBC and ROESY correlations of **1**.

Compound **1** was obtained as oil. The molecular formula C_24_H_35_NO_5_ with eight degrees of unsaturation was established by the positive mode quasi-molecular ion peaks at *m*/*z* 418.2592 for [M + H]^+^ (calcd. 418.2593 for C_24_H_36_NO_5_) and *m*/*z* 440.2398 for [M + Na]^+^ (calcd. 440.2413 for C_24_H_35_NO_5_Na) in combination with its NMR data. The ^1^H NMR together with heteronuclear single quantum coherence (HSQC) spectra revealed the presence of five methyl groups [δ_H_ 0.90 (*d*, *J* = 2.5 Hz), 0.91 (*d*, *J* = 2.5 Hz), 1.21 (*d*, *J* = 7.0 Hz), 1.42 (s), 1.76 (s)], two olefinic protons [δ_H_ 5.39 (s), 6.04 (*d*, *J* = 11.0 Hz)], four oxygenated methine protons [δ_H_ 2.82 (s), 3.78 (*m*, overlapped), 3.80 (*m*, overlapped), 4.40 (*d*, *J* = 1.9 Hz)], three methylene protons including two diastereotopic methylenes [δ_H_ 1.29 (m), 2.29/2.10 (m), 1.76/1.63 (*m*)] and five aliphatic methine protons [δ_H_ 1.56 (*m*), 2.60 (*m*), 2.64 (*m*), 3.02 (*d*, *J* = 11.5 Hz), 3.10 (*d*, *J* = 10.0 Hz)]. In addition, five quaternary carbons were extracted from the heteronuclear multiple bond correlation (HMBC) spectrum, including one keto carbonyl (δ_C_ 208.0), one amide carbonyl (δ_C_ 174.6), two *sp*^2^-quaternary carbons (δ_C_ 135.6, 140.6) and one *sp*^3^-quaternary carbon at δ_C_ 68.4 (Table 1). These features supported the characteristic aspochalasin skeleton [25]. A detailed NMR data analysis and further literature search showed the close similarity of **1** to the previously isolated compound aspochalasin H [26] (Table 1). Due to the obvious ^13^C chemical shift difference in the stereocenters (C-17, C-18, C-19 and C-20) of **1** from the NMR data of aspochalasin H (δ_C_ aspochalasin H/compound **1**: C-17, 72.9/70.8; C-18, 78.4/73.4; C-19, 61.7/60.5; C-20, 54.7/52.2), we concluded that **1** was a stereoisomer of aspochalasin H, probably with a different diol orientation. 2D ROESY provided important information on the proton signals arising from each proton through the space, even though they were not bonded to each other. The ROESY experiment showed correlations between H-4 and H-8, between H-4 and H-10, and between H-3 and H-11, which were identical to the isoindolone moiety of the reported cytochalasans, especially aspochalasin H [26], except H-17, H-18, H-19 and H-20. The small coupling constant (*J* = 1.9 Hz) between H-19 and H-20 indicated a *trans* epoxide, which was the same as that in aspochalasin H [26]. Moreover, the ROESY and NOE correlations between H-19 and H-17 and between H-13 and H-17 revealed an alpha (α) orientation of H-17 (Figure 2) and probably a beta (β) orientation of H-18. If H-17 were in a beta (β) orientation, it would be impossible to have a NOE correlation between H-13 and H-17.

To confirm the absolute configuration of compound **1**, advanced computational quantum chemical calculations were carried out. The NMR of eight stereoisomers (Supplementary Information, Table S1) were calculated, among which the calculated NMR data of the 17*R*, 18*S*, 19*S*, 20*R* and 17*R*, 18*S*, 19*R*, 20*R* isomers matched those of the experimental data of **1** better than the other six isomers. Electronic Circular Dichroism (ECD) is a very sensitive and nondestructive experimental technique, which may provide a powerful approach to the determination of the absolute configurations of the molecule [27]. Both the experimental ECD of **1** and the calculated ECDs of the 17*R*, 18*S*, 19*S* and 20*R* isomer together with the 17*R*, 18*S*, 19*R* and 20*R* isomer showed a strong positive Cotton effect at 225 nm (Figure S9). We then carried out a conformation analysis (Figure S10). In all the main stable conformers of 17*R*, 18*S*, 19*S,* 20*R* and 17*R*, 18*S*, 19*R*, 20*R* isomers, H-19/H-17 were in the axial/equatorial orientations in the 11-member ring, respectively. H-18 was equatorial in the 17*R*, 18*S*, 19*S*, 20*R* isomer and axial in the 17*R*, 18*S*, 19*R*, 20*R* isomer. Since H-17 and H-18 were overlapped in CDCl_3_, we collected ^1^H NMR of **1** in DMSO-*d*_6_. The ^1^H NMR spectrum of **1** in DMSO-*d*_6_ showed that H-19 was still a singlet and H-18 was a doublet with a coupling constant of about 10 Hz. Only H-18 in equatorial form (17*R*, 18*S*, 19*S*, 20*R*) could meet the H-H coupling requirement of the experimental results. Hence, the absolute configuration of **1** was determined to be 17*R*, 18*S*, 19*S*, 20*R*.

The other isolated known metabolites from *Aspergillus* sp. FT1307 were structurally characterized by comparing their NMR and optical rotation data with the literature and were identified as aspochalasin P (**2**) [28], alatinone (**3**) [29], dimerumic acid (**4**) [30], NBRI-16716B (*N*,5-dihydroxy-*N*-(3-5-(3-(N-hydroxyacetamido)propyl)-3,6-dioxopiperazin-2-yl)propyl)-3-methylpent-2-enamide)) (**5**) [31], α-11-hydroxyurvularine (**6**) [32], β-11-hydroxycurvularine (**7**) [32], β-11-methoxy curvularine (**8**) [32], 12-keto-10,11-dehydrocurvularine (**9**) [33] and WIN66306 (**10**) [34].

All the isolated compounds were tested for their antibacterial activity against pathogenic *Staphylococcus aureus* ATCC12600 (Gram-positive), *Bacillus subtilis* ATCC6633 (Gram-negative) and methicillin-resistant *S. aureus* ATCC43300 (MRSA). Compounds **2**, **3**, **8** and **9** showed weak activity in the range of 40 to 80 μg/mL (Table 2). Further, structural features of aspochalasins and their contribution to the bioactivity have been previously studied [35]. Flashner and co. [35] showed that the α,β-ketounsaturation in the 11-membered macrolide ring was responsible for antibacterial inhibitory activity, specifically against Gram-positive pathogenic bacteria [35].

Compounds **1**–**10** were also evaluated for their antiproliferative activity against MCF-7 (Human breast cancer cell line), T46D (Human breast cancer cell line) and A2780 (Human ovarian cancer cell line). Only compounds **2** and **8** showed a mild antiproliferative activity against the A2780 ovarian cancer cell line with IC_50_ values of 17.29 µM and 11.76 µM, respectively. Compounds **1**–**10** were further tested against an immortalized human vascular endothelial cell (EA.hy926) for their cytotoxicity effect, and they did not show any significant inhibition against EA.hy926 at the tested concentrations (50 μM).

## 3. Materials and Methods

### 3.1. General Experimental Procedures

Optical rotations, CD and IR spectra were measured with a Rudolph Research analytical AutoPol automatic polarimeter (Rudolph Research Analytical, Hackettstown, NJ, USA), JASCO J-815 CD (Jasco Corporation, Tokyo, Japan) and Thermo Scientific Nicolet iS10 IR spectrometer (Thermo Fisher Scientific, Madison, WI, USA), respectively. The structure characterizations of all the compounds were based on 1D NMR (^1^H, ^13^C) and 2D NMR (COSY, HSQC, HMBC, 1D-NOE and ROESY) data, recorded on a Bruker AM-400 spectrometer (Bruker BioSpin AG, Faellanden, Switzerland). An Agilent 6530 Accurate-Mass Q-TOF LC-MS spectrometer (Agilent Technologies, Waldbronn, Germany) was used to record high-resolution mass spectra. Preparative RP-HPLC was carried out on an Ultimate 3000 chromatographic system (Agilent Technologies, Waldbronn, Germany) with a Phenomenex preparative column (Phenyl-hexyl, 5 μ, 100 × 21.2 mm) and semipreparative RP-HPLC on an Ultimate 3000 chromatographic system (Agilent Technologies, Waldbronn, Germany) with a Phenomenex semipreparative column (C_18_, 5 μ, 250 × 10 mm), connected to a Dionex Ultimate 3000 DAD detector (Agilent Technologies, Waldbronn, Germany) (detected at 210, 254, 320 and 365 nm) and a Dionex Ultimate 3000 automated fraction collector. All solvents were HPLC grade. Diaion HP-20 (Alfa Aesar, Japan) was used to run the open-column chromatography.

### 3.2. Sample Collection and Fungi Isolation

Healthy and disease-free leaves from a plant were collected from Big Island, Hawai’i (155°3′15.51″ W 19°43′53.63″ N) in February 2020, and they were morphologically identified as *Heliotropium* sp. (family Boraginaceae). The voucher specimen was deposited at Daniel K. Inouye College of Pharmacy, University of Hawai’i at Hilo, HI, USA (Code: MQ21-2020). Leaves were washed thoroughly with running tap water and surface-sterilized in 70% ethanol for 1 min, in sterile distilled water for 1 min, in 2.5% sodium hypochlorite (NaOCl) for 1 min and again in sterile distilled water. The procedure was repeated three times to ensure the removal of surface-dwelling microorganisms. The sterilized samples were left on sterile filter papers for drying, then cut into small segments, placed on potato dextrose agar (PDA) and incubated at room temperature for 7–14 days. The growing fungi were removed before seven days to ensure the isolation of endophytic fungi. After the incubation, six morphologically different fungi were isolated and serially sub-cultured to potato dextrose agar (PDA)-containing Petri dishes until pure colonies were formed. Pure strains were maintained at −80 °C in 50% glycerol and deposited at Daniel K. Inouye College of Pharmacy, University of Hawai’i at Hilo, HI, USA (Code: MQ-FT1307-2020).

### 3.3. Molecular Identification of the Endophytic Fungi

The strain FT1307 was grown on liquid potato dextrose broth (PDB) media for 28 days, and the mycelium was harvested under aseptic conditions. About 300 μg of the mycelium was crushed using liquid nitrogen into fine powder. Then, DNA extraction, amplification and sequencing of the Internal Transcribe Spacer regions using the primers ITS1 and ITS4 were carried out as described in our previously published articles [12,13,14,15,16,17,18,19,20]. The BLAST search indicated that the sequence of the ITS region had a 98% similarity score between two *Aspergillus* species. Hence, strain FT1307 was unable to be identified to the species level. Therefore, FT1307 was identified as the unknown *Aspergillus* sp., and the sequence was deposited in the NCBI Gene Bank under Accession No. MW774248.

### 3.4. Fermentation and Extraction

Equal size agar plugs with the mycelium of the pure FT1307 fungus grown on PDA media for five days was aseptically transferred to liquid broth media-containing 1-L conical flasks (350 mL × 65 for the large scale; 350 mL × 3 for the small scale). The liquid broth media consisted of mannitol 20 g, sucrose 10 g, monosodium glutamate 5 g, KH_2_PO_4_ 0.5 g, MgSO_4_·7H_2_O 0.3 g and 3 g of yeast extract dissolved in 1 L of distilled water (pH of the liquid media adjusted to 6.5 before sterilization). Then, the media flasks were sterilized for 15 min at 121 °C. The inoculated flasks were fermented for 28 days at 24 °C under static conditions. After the completion of the fermentation period, extraction was carried out as described in our previous publications [18,19] with slight modifications. Aqueous mycelium and broth media treated with Diaion HP-20 resins were loaded into an open column and separated using different concentrations of MeOH/H_2_O systems (10, 50, 90 and 100% MeOH) as the eluent. The 90% and 100% fractions shared a similar RP-HPLC profile. Hence, both fractions were combined (3.5 g) and subjected to further purification.

### 3.5. Isolation of Fungal Secondary Metabolites

Combined fractions were initially purified by using preparative RP-HPLC (Phenyl-hexyl column, 100 × 21.20 mm, 5 μ, 8 mL/min) eluted with 40−100% MeOH/H_2_O in 20 min to yield 26 subfractions (SFr 1−26). SFr 4 and 5 were combined (170 mg) and purified using semipreparative RP-HPLC (30% isocratic MeOH/H_2_O, 1.0% formic acid, 3.0 mL/min for 20 min) over a C18 column to furnish compound **4** (*t_R_* 9.8 min, 2.5 mg). SFr 8 (150 mg) was purified using semipreparative RP-HPLC (40% isocratic MeOH/H_2_O, 1.0% formic acid, 3.0 mL/min for 20 min) over a C18 column to afford compounds **5** (*t_R_* 11.8 min, 2.0 mg) and **7** (*t_R_* 15.5 min, 2.0 mg). SFr 10 (170 mg) was purified using semipreparative RP-HPLC (50% isocratic MeOH/H_2_O, 1.0% formic acid, 3.0 mL/min for 20 min) over a C18 column to afford compounds **6** (*t_R_* 12.4 min, 2.0 mg) and **3** (*t_R_* 15.5 min, 2.0 mg). SFr 11–14 were combined due to a low yield (120 mg) and purified using semipreparative RP-HPLC (60% isocratic MeOH/H_2_O, 1.0% formic acid, 3.0 mL/min for 20 min) over a C18 column to afford compounds **8** (*t_R_* 8.8 min, 1.5 mg) and **9** (*t_R_* 18.2 min, 1.5 mg). SFr 16 was (100 mg) purified using semipreparative RP-HPLC (60% isocratic MeOH/H_2_O, 1.0% formic acid, 3.0 mL/min for 20 min) over a C18 column to afford compound **10** (*t_R_* 20.1 min, 5.0 mg). SFr 17 was (150 mg) purified using semipreparative RP-HPLC (70% isocratic MeOH/H_2_O, 1.0% formic acid, 3.0 mL/min for 20 min) over a C18 column to afford compounds **1** (*t_R_* 13.2 min, 2.0 mg) and **2** (*t_R_* 17.9 min, 5.0 mg).

**Aspochalasin H1** (**1**): oil; [α]^25^ = 25 (c = 0.12, MeOH). UV (MeOH): λ_max_ (log ε) = 208 (2.90) nm. IR (KBr): ν_max_ = 3327, 2945, 2835, 1652, 1452, 1114, 1016 cm^−1^. ^1^H and ^13^C NMR: See Table 1. HR-ESI-MS *m*/*z* 418.2592 [M + H]^+^ (calc. 418.2593) 440.2398 [M + Na]^+^ (calc. 440.2413) for C_24_H_35_NO_5_.

### 3.6. Computational Methods

The ECD and NMR computational calculations were carried out as previously described in our publications [36,37].

### 3.7. Antibacterial Assay

The assay was carried out according to the EUCAST guidelines with modifications. Bacteria were grown on agar plates containing tryptic soy agar (TSA) or Brain Heart Infusion (BHI)] for 24 h at 37 °C and then added to the corresponding broth medium (TSB for *S. aureus* and methicillin-resistant *S. aureus*, and BHI for *B. subtilis*). After incubation at 37 °C overnight, cultures were diluted with TSB or BHI media to obtain an absorbance of 0.1 at OD_600_ (McFarland 0.5 and 5 × 10^5^ CFU/mL). The bacteria-containing media (100 μL) were then added to each well of 96-well plates, containing each compound dissolved in 0.5% DMSO solution (100 μL) in TSB or BHI media. Then, the 96-well plates were incubated at 37 °C for 18 h. After 18 h, twenty microliters of resazurin dye (0.015%) were then added to each well and further incubated for 2–3 h at 37 °C. The color change (blue to pink) was visually observed. 1% DMSO was used as the negative control, whereas chloramphenicol was used as the positive control, which was active against *S. aureus*, multidrug-resistant *S. aureus* and *B. subtilis* with MIC values ranging from 5 µg/mL to 10 µg/mL. All experiments were performed in triplicate. The compounds were tested from a 0.625 to 80 µg/mL concentration range.

The Minimum Inhibitory Concentration (MIC) is defined as the lowest concentration of a drug or compound/s that inhibits the visible growth of a bacterial organism after a specific incubation time [38].

### 3.8. Cell Viability Assay

An MTT assay was performed to estimate the viability of two human breast cancer cell lines (MCF-7 and T46D), one human ovarian cancer cell line (A2780) and one healthy human cell line (immortalized human vascular endothelial cell/EA.hy926). Cells (1 × 10^4^ per well) were seeded in 96-well plates, cultured at 37 °C in a 5% CO_2_ incubator for 24 h, and then incubated with or without the compounds for 24 h. The culture medium was carefully removed, and 200 μL of DMSO was added per well to dissolve the formed precipitate. Plates were shaken for 10 s, and the absorbance was measured at a wavelength of 570 nm on a microplate reader (BIO-TEK instruments, Inc., Winooski, VT, USA).

## 4. Conclusions

In conclusion, ten compounds (**1**–**10**) including a new cyclic aspochalasin H1 (**1**) were isolated from a Hawaiian plant-associated endophytic fungus, *Aspergillus* sp. FT1307. Compound **1** is a stereoisomer of aspochalasin H with an adjacent *trans*-diol configuration. Compounds **1**–**10** were evaluated for their antibacterial activity against Gram-positive and Gram-negative bacteria as well as for their antiproliferative activities against A2780, MCF-7 and T46D cell lines. Compounds **2**, **3**, **8** and **9** showed moderate antibacterial activity against gram-positive bacteria, and compounds **2** and **8** also showed antiproliferative activity against the A2780 ovarian cancer cell line. The finding of the current study indicates that the Hawaiian environment is an arsenal of fungi that can produce new and bioactive compounds.

## Figures and Tables

**Table 1:** ^13^C (100 MHz) and ^1^H (400 MHz) NMR assignments for compound **1** in CDCl_3_.

No.	*δ*_C_	*δ*_H_ (Multiplicity, *J* in Hz)	*δ*_C_ [a]	*δ*_H_ (Multiplicity, *J* in Hz) [a]
**1**	174.6	-	174.6	-
**2**-NH	-	6.32 (*brs*)	-	6.06 (*brs*)
**3**	51.6	3.10 (*d*,10.0)	51.7	3.08 (*dt*, 10.0, 3.5)
**4**	52.9	2.64 (*m*)	52.8	2.64 (*d*, 3.5)
**5**	35.4	2.60 (*m*)	35.4	2.58 (*m*)
**6**	140.6	-	140.7	-
**7**	125.6	5.39 (*s*)	125.5	5.38 (*m*)
**8**	43.8	3.02 (*d*,11.5)	44.1	3.02 (*d*, 11.0)
**9**	68.4	-	67.4	-
**10**	48.8	1.29 (*m*)	48.6	1.29, 1.19 (*m*)
**11**	13.7	1.21 (*d*, 7.0)	13.7	1.19 (*d*, 7.0)
**12**	20.1	1.76 (*s*)	20.1	1.75 (*s*)
**13**	125.2	6.04 (*d*, 11.0)	124.6	5.98 (*d*, 11.0)
**14**	135.6	-	136.0	-
**15**	38.8	2.29, 2.10 (*m*)	38.9	2.25, 2.11 (*m*)
**16**	31.0	1.76, 1.63 (*m*)	29.3	2.12, 1.53 (*m*)
**17**	70.8	3.78 (*m, overlapped*)	72.9	4.04 (*s*)
**18**	73.7	3.80 (*m, overlapped*)	78.4	3.37 (*d*, 7.0)
**19**	60.5	2.82 (*s*)	61.7	2.62 (*dd*, 7.0, 1.5)
**20**	52.2	4.40 (*d*, 1.9)	54.7	4.19 (*d*, 1.5)
**21**	208.0	-	205.6	-
**22**	25.2	1.56 (m)	25.2	1.53 (*m*)
**23**	23.7	0.91 (*d*, 2.5)	23.6	0.90 (*d*, 6.0)
**24**	21.4	0.90 (*d*, 2.5)	21.2	0.88 (*d*, 6.0)
**25**	15.5	1.42 (*s*)	15.5	1.42 (*s*)

[a] Reference ^13^C (125 MHz) and ^1^H (500 MHz) NMR data for aspochalasin H in CDCl_3_ [26]. ^13^C NMR data of **1** was extracted from the HSQC and HMBC spectra.

**Table 2:** MIC (μg/mL) of the tested compounds.

Compound No.	*Staphylococcus aureus* ATCC12600	Methicillin-Resistant *S. aureus* ATCC43300	*Bacillus subtilis* ATCC6633
**2**	*	*	40
**3**	40	40	40
**8**	*	*	80
**9**	40	40	80
Chloramphenicol	5	5	10

**1**, **4**–**7** and **10** were not active at 80 μg/mL; * MIC ≥ 80 μg/mL.

## Supplementary Materials

The following are available online. 1D and 2D NMR, CD spectra of compound **1**.

## Sample Availability

Samples of the compounds are not available.
