# Supplementary material for: Aspochalasin H1: A New Cyclic Aspochalasin from Hawaiian Plant-Associated Endophytic Fungus Aspergillus sp. FT1307
Source: Molecules. 2021 Jul 12;26(14):4239. doi: 10.3390/molecules26144239 (PMC8307934; doi:10.3390/molecules26144239)
Supplement: Supplementary file 1 [file molecules-26-04239-s001.zip › molecules-1283029-supplementary.pdf]

Supplementary Data

Aspochalasin H1: A New Cyclic Aspochalasin from Hawaiian Plant-associated Endophytic Fungus *Aspergillus* sp. FT1307

Mallique Qader [1,†], KH Ahammad Uz Zaman [1,†], Zhenquan Hu [2,3], Cong Wang [1,4], Xiaohua Wu [1] and Shugeng Cao [1,*]

1 Department of Pharmaceutical Sciences, Daniel K. Inouye College of Pharmacy, University of Hawai'i at Hilo, Hilo, Hawai'i 96720, USA; mqader@hawaii.edu (M.Q.); kzaman@hawaii.edu (K.A.U.Z.); congwang@hawaii.edu (C.W.); xiaohua3@hawaii.edu (X.W.)
2 Warshel Institute for Computational Biology, The Chinese University of Hong Kong, Shenzhen 518172, China; huzhenquan@cuhk.edu.cn (Z.H.)
3 School of Chemistry and Materials Science, University of Science and Technology of China, Hefei 230026, China
4 Guangxi Key Laboratory of Chemistry and Chemical Engineering of Forest Products, School of Chemistry for Nationalities, Nanning 530006, China.
† These authors contributed equally to this work.
* Correspondence: scao@hawaii.edu; Tel.: 1-808-981-8017

Figures

Figure S1: HR-ESI-MS spectrum of **1** 2
Figure S2: ^{1}H NMR spectrum of **1** (400 MHz, DMSO-d_6, 298 K) 2
Figure S3: ^{1}H NMR spectrum of **1** (400 MHz, $CDCl_3$, 298 K) 3
Figure S4: ^{1}H-^{1}H COSY spectrum of **1** (400 MHz, $CDCl_3$, 298 K) 3
Figure S5: HSQC spectrum of **1** (400 MHz, $CDCl_3$, 298 K) 4
Figure S6: ROESY spectrum of **1** (400 MHz, $CDCl_3$, 298 K) 4
Figure S7: HMBC spectrum of **1** (400 MHz, $CDCl_3$, 298 K) 5
Figure S8: IR spectra of **1** 5
Figure S9: Experimental and calculated ECD spectra of **1** 6
Figure S10: Conformation analysis of **1** (17R,18S,19S,20R and 17R,18S,19R,20R) 7

Tables

Table S1: NMR calculations of **1** 8

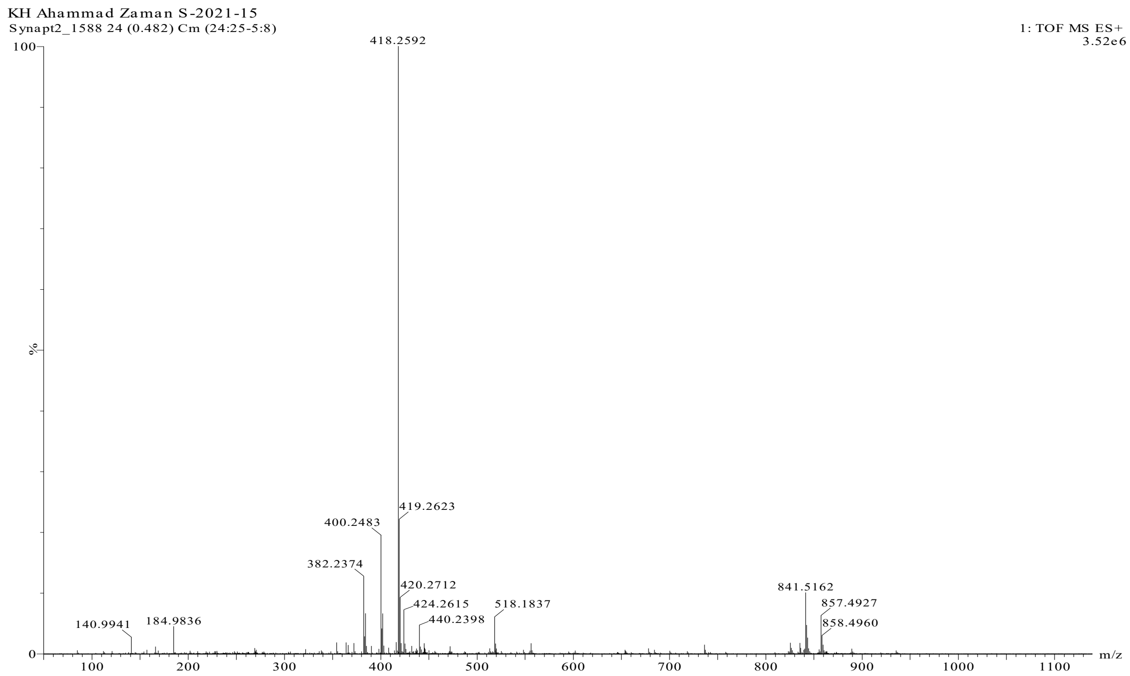

Figure S1: HR-ESI-MS spectrum of **1**

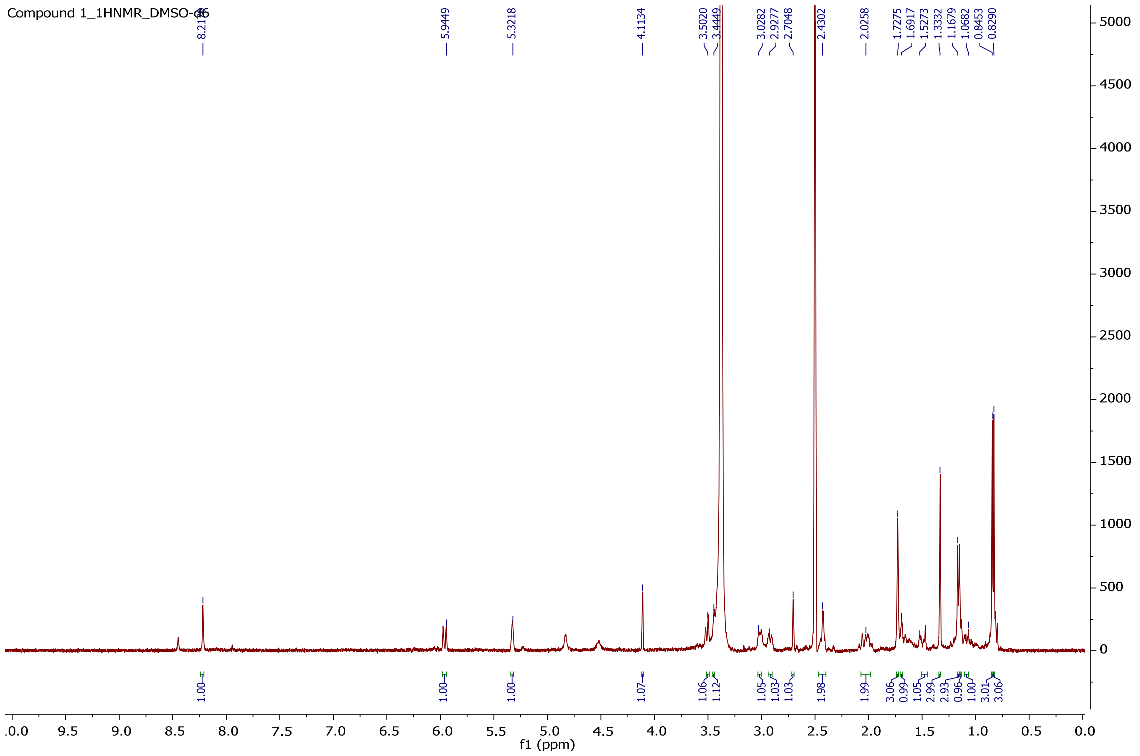

Figure S2: ^{1}H NMR spectrum of 1 (400 MHz, DMSO-d_6, 298 K)

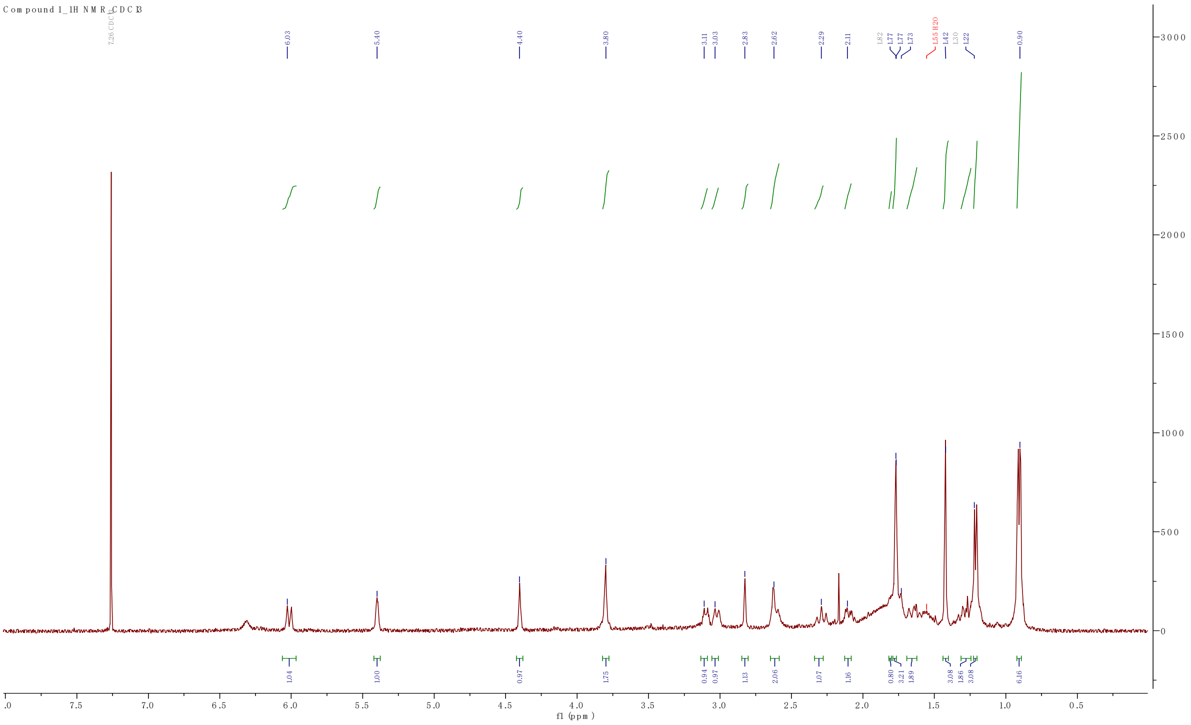

Figure S3: ^1H NMR spectrum of 1 (400 MHz, $CDCl_3$, 298 K)

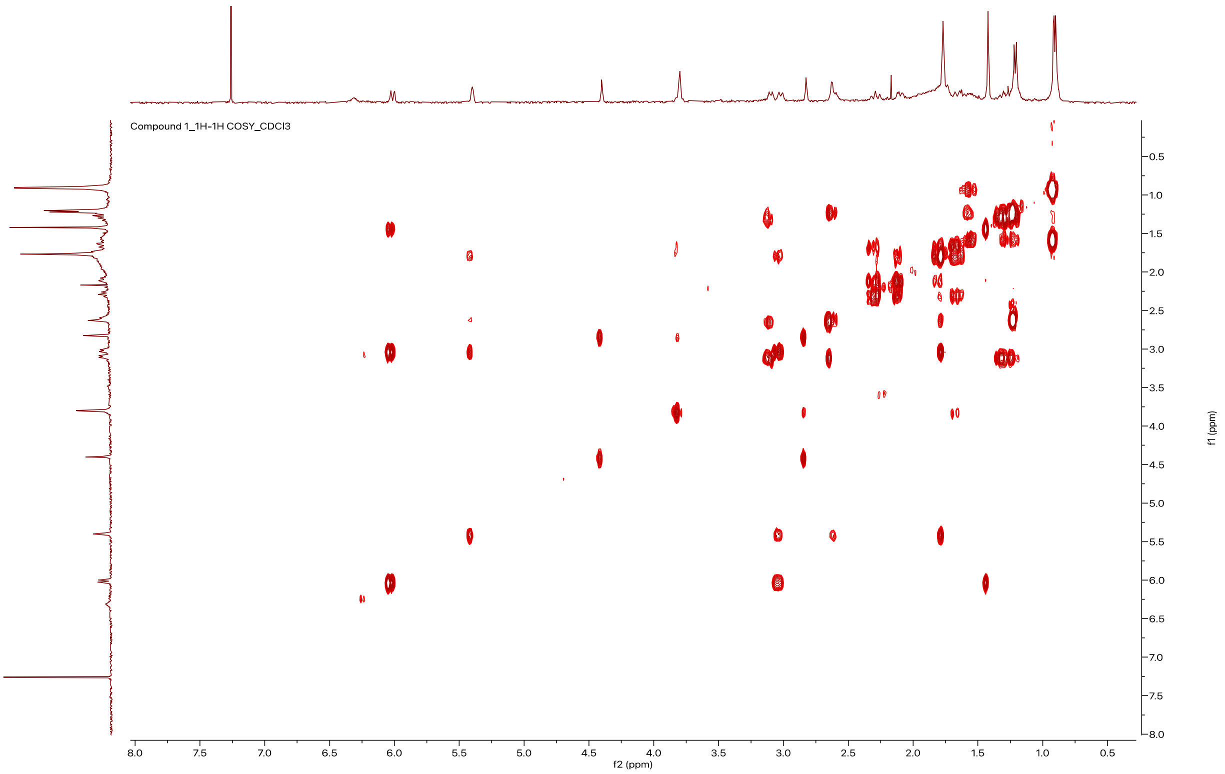

Figure S4: ^1H-^1H COSY spectrum of **1** (400 MHz, $CDCl_3$, 298 K)

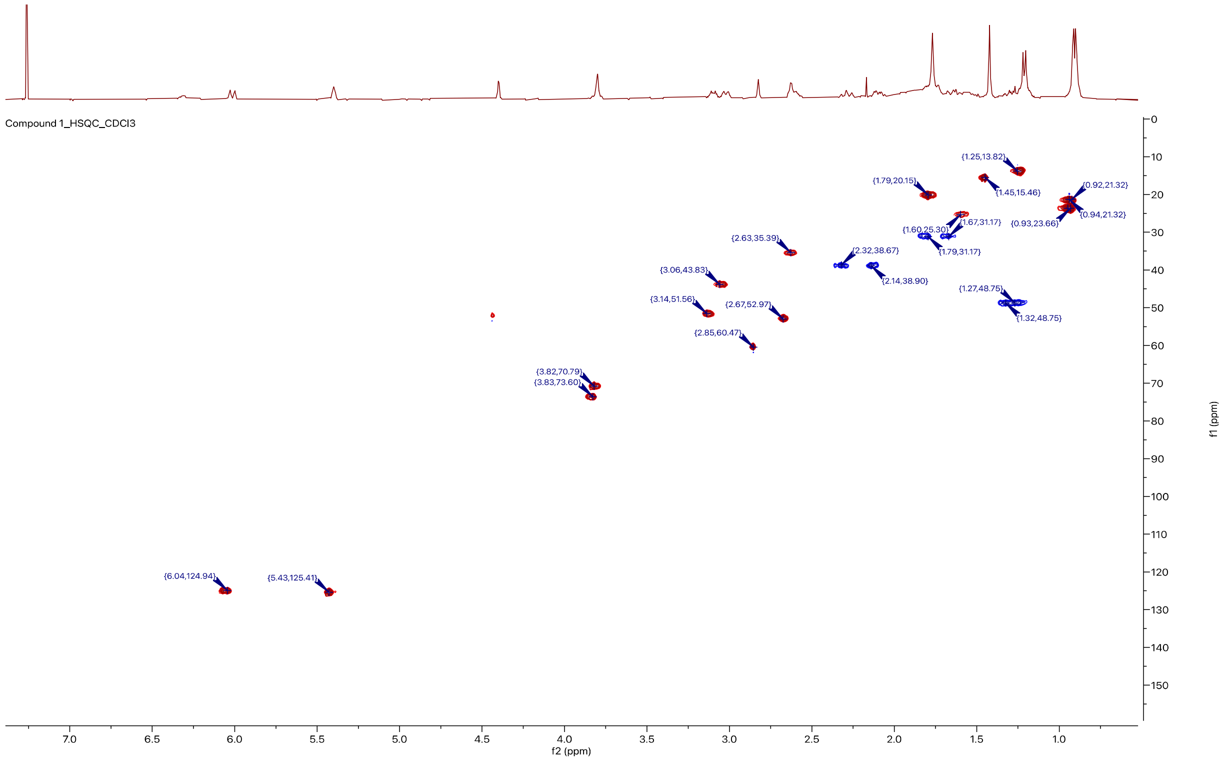

Figure S5: HSQC spectrum of **1** (400 MHz, CDCl3, 298 K)

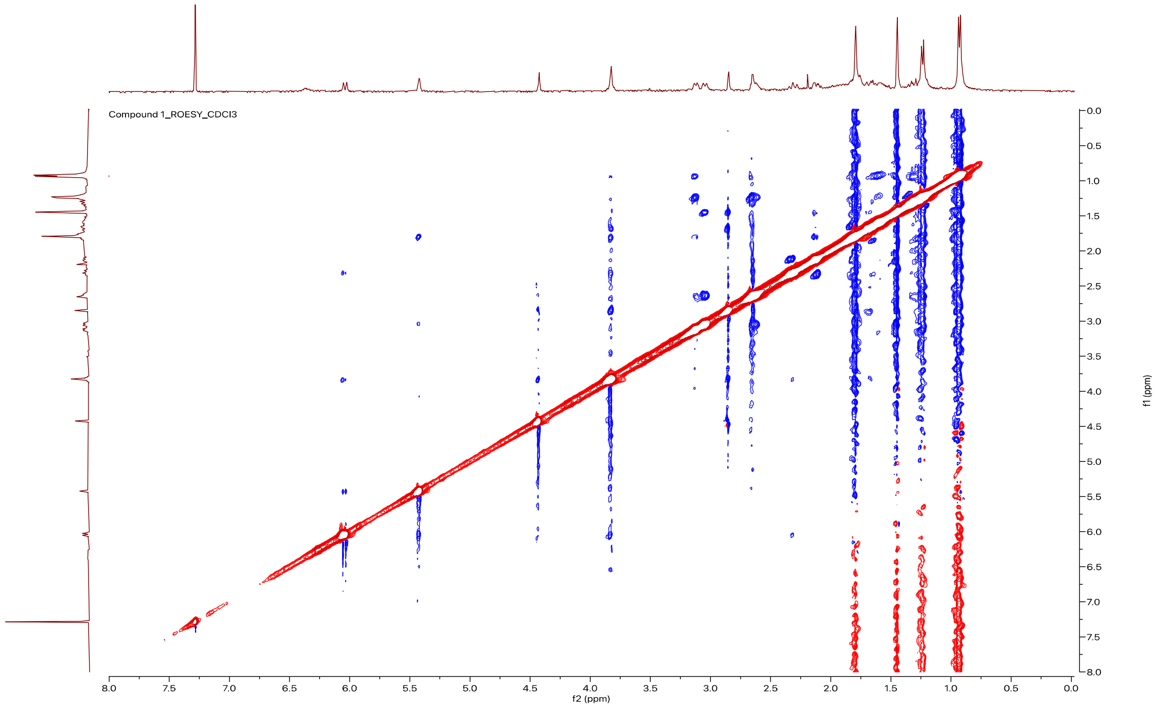

Figure S6: ROESY spectrum of **1** (400 MHz, $CDCl_3$, 298 K)

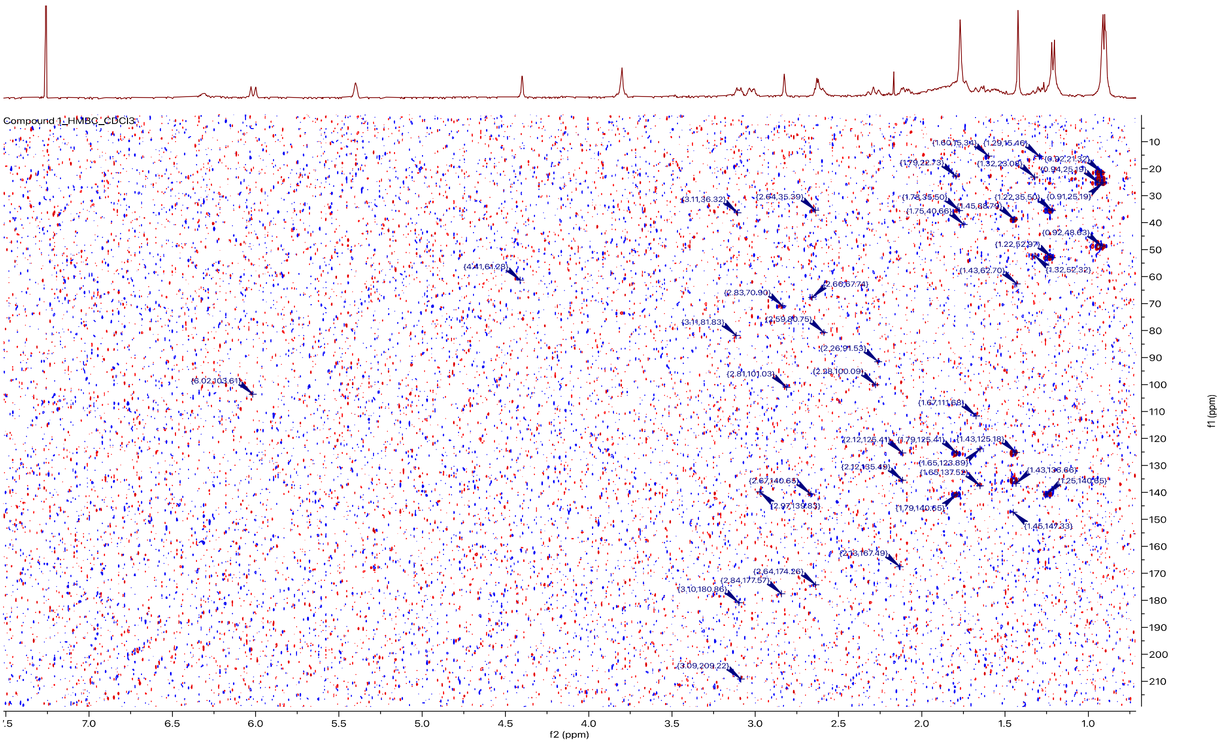

Figure S7: HMBC spectrum of **1** (400 MHz, $CDCl_3$, 298 K)

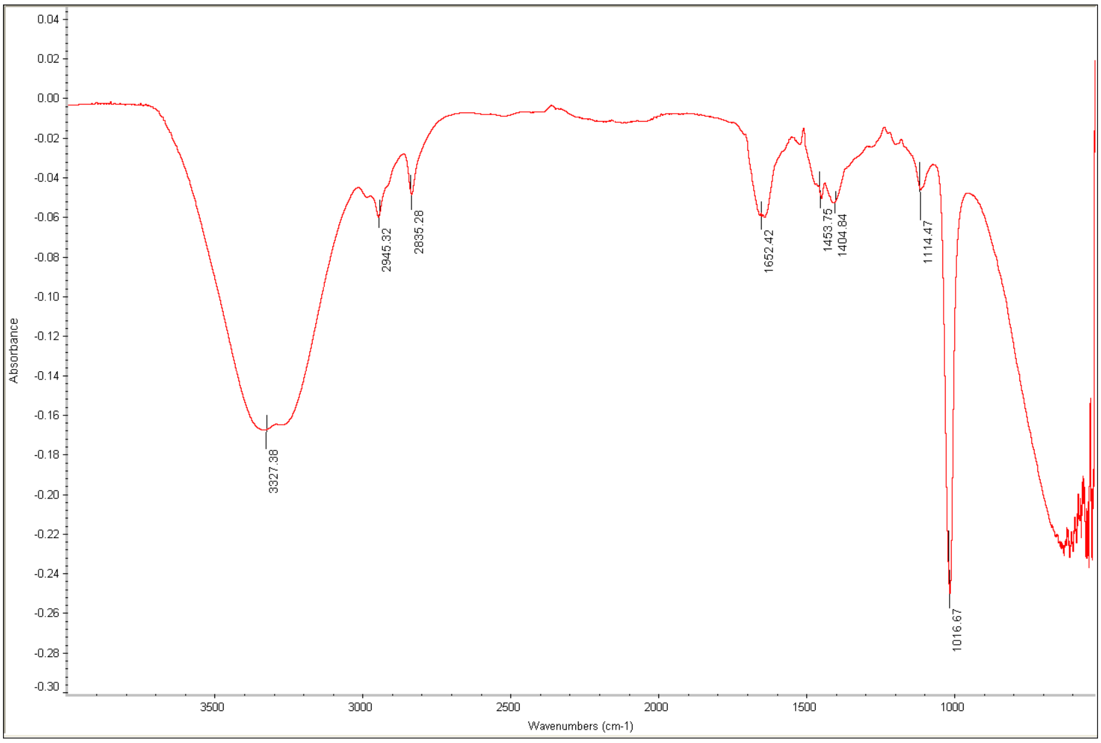

Figure S8: IR spectrum of **1**

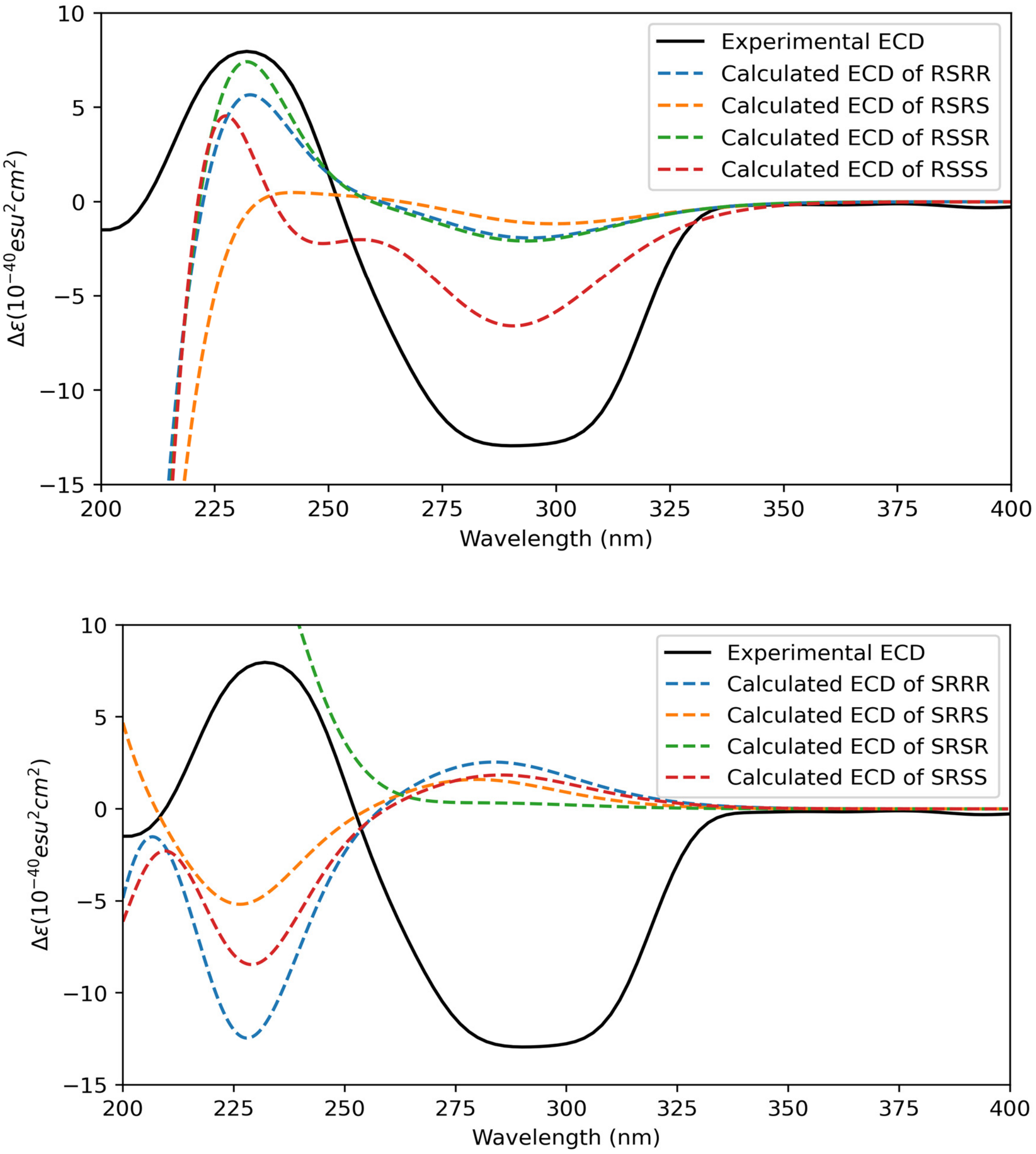

Figure S9: ECD spectra of **1**

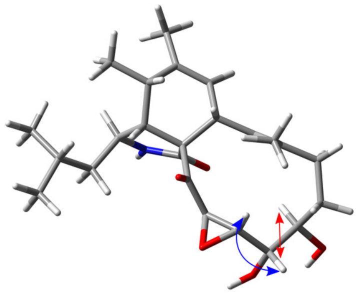
RSSR-1 45.4%

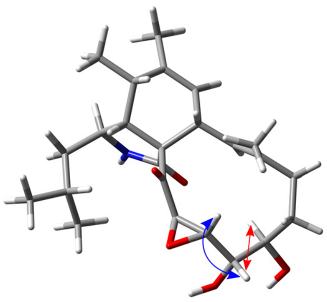
RSSR-2 22.5%

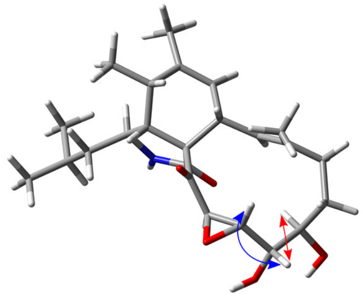
RSSR-3 20.3%

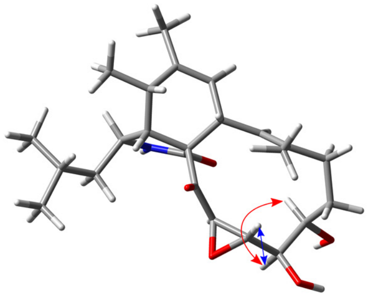
RSRR-1 31.1%

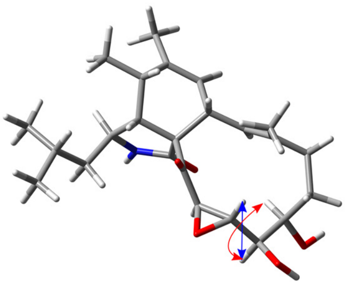
RSRR-2 18.2%

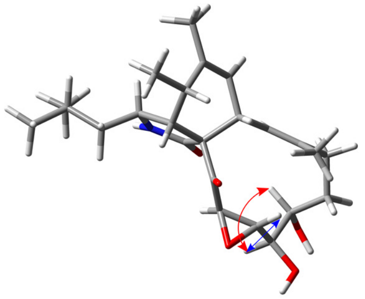
RSRR-3 15.4%

Figure S10: Conformation analysis of **1** (17R,18S,19S,20R and 17R,18S,19R,20R)

Table S1: NMR calculations of **1**

	Experimental data		RSRR		RSRS		RSSR		RSSS		SRRR		SRRS		SRSR		SRSS	
**C/H No**	**δC**	**δH**	**δC**	**δH**	**δC**	**δH**	**δC**	**δH**	**δC**	**δH**	**δC**	**δH**	**δC**	**δH**	**δC**	**δH**	**δC**	**δH**
**1**	174.6		173.5		173.4		173.7		173.1		172.6		172.2		173.5		174.1	
**3**	51.6	3.10	54.9	3.19	53.9	5.47	52.6	3.15	54.1	5.47	54.2	5.42	52.9	5.34	52.8	5.45	52.8	5.50
**4**	52.9	2.64	56.2	2.96	55.7	2.97	57.2	2.71	53.8	2.99	59.4	3.45	54.9	3.41	59.3	3.60	59.4	3.68
**5**	35.4	2.60	38.6	2.52	39.4	4.67	39.3	2.55	39.2	4.88	38.4	3.67	39.0	4.07	39.6	3.46	40.0	3.49
**6**	140.6		146.5		146.8		146.4		146.7		145.4		147.4		146.1		145.8	
**7**	125.6	5.39	126.2	5.44	127.3	2.95	126.7	5.44	127.0	2.99	129.1	2.97	126.5	3.23	127.9	2.39	128.0	2.30
**8**	43.8	3.02	49.0	3.13	48.4	2.48	49.0	3.12	48.5	2.45	39.8	2.45	42.0	2.42	40.6	2.58	41.5	2.60
**9**	68.4		73.0		72.4		72.7		73.6		71.7		73.3		71.5		71.2	
**10**	48.8	1.29	47.5	1.26	49.3	1.35	49.2	1.20	46.5	1.30	47.3	1.59	46.4	1.62	46.6	1.63	47.6	1.51
**11**	13.7	1.21	11.9	1.29	13.3	1.25	12.3	1.28	12.6	1.26	13.7	1.18	14.5	1.28	13.9	1.23	13.4	1.22
**12**	20.1	1.76	19.2	1.80	20.0	2.24	19.4	1.83	19.6	2.27	19.7	2.17	19.9	2.27	20.0	2.26	19.9	2.31
**13**	125.2	6.04	127.1	6.27	127.1	2.07	127.9	6.25	126.5	1.96	127.8	2.27	132.2	2.10	129.0	2.30	128.8	1.65
**14**	135.6		141.1		143.3		140.4		144.0		141.5		142.2		139.7		142.1	
**15**	38.8	2.10,2.29	40.4	2.17,2.34	36.8	1.97,2.49	40.1	2.13,2.36	37.3	1.63,2.54	42.1	2.28,2.81	35.6	1.91,2.45	40.4	2.06,2.42	31.6	2.19,2.65
**16**	31.0	1.63,1.76	32.7	1.53,2.29	36.2	1.83,3.67	33.4	1.70,1.79	38.5	2.50,3.61	31.9	1.47,3.94	39.5	1.82,3.03	38.6	1.72,3.51	37.5	1.939,3.96
**17**	73.7	3.80	74.6	3.74	72.1	3.23	76.7	3.74	75.4	4.31	74.2	4.12	67.8	3.78	72.1	2.74	78.6	2.92
**18**	70.8	3.78	80.8	3.37	78.3	1.86	74.1	3.71	75.5	1.80	76.9	1.73	72.4	1.86	79.0	1.99	75.0	1.83
**19**	60.5	2.82	64.6	2.47	65.2	6.48	63.6	2.67	64.1	6.48	63.3	6.32	62.8	6.84	65.2	6.87	62.3	7.25
**20**	52.2	4.40	56.2	4.17	57.5	2.74	54.6	4.31	51.8	2.99	58.8	3.08	57.8	3.12	59.5	2.81	61.7	3.18
**21**	208.0		210.6		208.9		211.4		210.1		211.7		209.1		212.7		212.6	
**22**	25.2	1.56	30.4	1.64	31.5	1.69	29.8	1.62	28.6	1.55	31.8	1.74	31.1	1.71	27.5	1.53	29.1	1.60
**23**	23.7	0.91	21.9	1.00	21.7	0.96	22.0	0.89	20.5	0.92	21.4	1.00	19.1	1.03	23.2	0.95	19.0	0.95
**24**	21.4	0.90	23.9	0.96	21.0	0.97	20.9	0.97	23.8	0.92	21.3	1.01	22.3	1.06	20.3	0.96	23.1	0.98
**25**	15.5	1.42	14.3	1.49	17.0	1.60	14.3	1.47	19.2	1.64	14.4	1.79	17.6	1.82	14.6	1.79	18.2	1.81

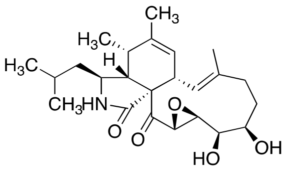
RSRR

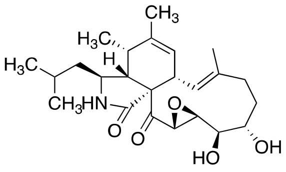
RSRS

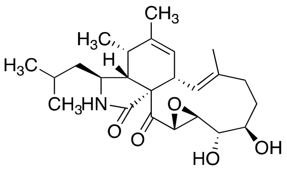
RSSR

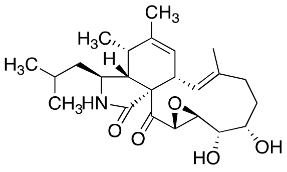
RSSS

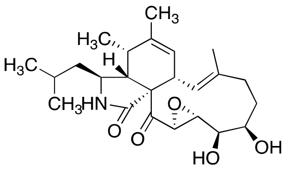
SRRR

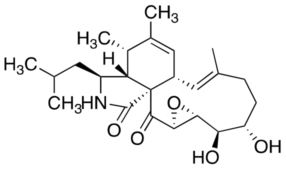
SRRS

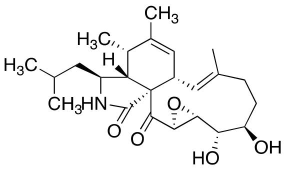
SRSR

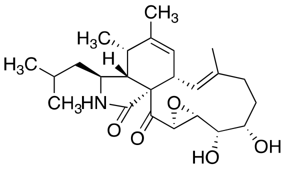
SRSS
